# Designing an Egocentric Video-Based Dashboard to Report Hand Performance Measures for Outpatient Rehabilitation of Cervical Spinal Cord Injury

**DOI:** 10.46292/sci23-00015S

**Published:** 2023-11-17

**Authors:** Adesh Kadambi, Andrea Bandini, Ryan D. Ramkalawan, Sander L. Hitzig, José Zariffa

**Affiliations:** 1 KITE – Toronto Rehabilitation Institute, University Health Network, Toronto, ON, Canada;; 2 Institute of Biomedical Engineering, University of Toronto, Toronto, ON, Canada;; 3 Health Science Interdisciplinary Center, Scuola Superiore Sant’Anna, Pisa, Italy;; 4 The Biorobotics Institute, Scuola Superiore Sant’Anna, Pisa, Italy;; 5 Department of Excellence in Robotics and AI, Scuola Superiore Sant’Anna, Pisa, Italy;; 6 Rehabilitation Sciences Institute, Temerty Faculty of Medicine, University of Toronto, Toronto, ON, Canada;; 7 St. John’s Rehab Research Program, Sunnybrook Research Institute, Sunnybrook Health Sciences Centre, Toronto, ON, Canada;; 8 Department of Occupational Science & Occupational Therapy, Temerty Faculty of Medicine, University of Toronto, Toronto, ON, Canada;; 9 Edward S. Rogers Sr. Department of Electrical and Computer Engineering, University of Toronto, Toronto, ON, Canada

**Keywords:** egocentric vision, hand function, home monitoring, spinal cord injury, upper limb rehabilitation, user-centred design, user stories

## Abstract

**Background:**

Functional use of the upper extremities (UEs) is a top recovery priority for individuals with cervical spinal cord injury (cSCI), but the inability to monitor recovery at home and limitations in hand function outcome measures impede optimal recovery.

**Objectives:**

We developed a framework using wearable cameras to monitor hand use at home and aimed to identify the best way to report information to clinicians.

**Methods:**

A dashboard was iteratively developed with clinician (*n* = 7) input through focus groups and interviews, creating low-fidelity prototypes based on recurring feedback until no new information emerged. Affinity diagramming was used to identify themes and subthemes from interview data. User stories were developed and mapped to specific features to create a high-fidelity prototype.

**Results:**

Useful elements identified for a dashboard reporting hand performance included summaries to interpret graphs, a breakdown of hand posture and activity to provide context, video snippets to qualitatively view hand use at home, patient notes to understand patient satisfaction or struggles, and time series graphing of metrics to measure trends over time.

**Conclusion:**

Involving end-users in the design process and breaking down user requirements into user stories helped identify necessary interface elements for reporting hand performance metrics to clinicians. Clinicians recognized the dashboard’s potential to monitor rehabilitation progress, provide feedback on hand use, and track progress over time. Concerns were raised about the implementation into clinical practice, therefore further inquiry is needed to determine the tool’s feasibility and usefulness in clinical practice for individuals with UE impairments.

## Introduction

Cervical spinal cord injury (cSCI) results in tetraplegia, the bilateral impairment of the upper and lower extremities. For individuals with tetraplegia, remaining function in the upper extremities (UEs) often replaces the functions of other parts of the body that no longer function (e.g., wheelchair propulsion replacing lower-limb locomotion).^1^ Therefore, functional use of the UEs is one of the primary determinants of independence and the top recovery priority for most individuals with cSCI.^2^,^3^ However, three factors related to the transition from inpatient to outpatient rehabilitation interfere with the optimal UE recovery: (1) patients being discharged too early from inpatient care (i.e., before achieving a plateau of neurorecovery) due to financial pressure on the healthcare system^4^-^6^; (2) an inability to effectively monitor patient recovery when patients return to communities away from specialized tertiary rehabilitation centres^7^; and (3) limited ability to determine whether motor improvements achieved in the clinics translate to increased hand use at home. This last point arises because current hand function outcome measures primarily capture the *capacity* domain of the International Classification of Functioning, Disability and Health (i.e., an individual’s ability to execute a task or action in an ideal or standardized environment) rather than the *performance* domain (i.e., an individual’s actual functioning in their real-life environment, taking into account both their abilities and the influences of environmental and personal factors).^8^ These factors create barriers to the optimal recovery of the UEs and do not allow planning interventions tailored to the patient’s needs.

To overcome these barriers, we recently developed a video-based algorithmic framework to monitor the hand use of individuals with cSCI living in the community,^9^ which combines egocentric cameras (i.e., cameras worn on the head) and state-of-theart computer vision algorithms to automatically detect hands, objects, and functional interactions between them.^10^-^16^ With egocentric vision, we can focus on the hands and objects being manipulated from the user’s perspective, as camera movements are based on their attention.^17^ This provides us with a functional context of hand use that other wearable sensors, such as accelerometers, inertial, and magnetic sensors, cannot achieve, as they only offer kinematic information of the UEs.^18^-^21^

Our results indicated that this framework can accurately detect functional hand-object interactions during unscripted activities recorded at home with an F1-score of 0.80.^9^,^22^ Simple outcome measures of hand use were then extracted from the frame-by-frame detection of functional interactions of both hands and validated against clinical assessments of hand function and independence such as the Upper Extremity Motor Score (UEMS), Graded Redefined Assessment of Strength, Sensibility and Prehension (GRASSP), and Spinal Cord Independence Measure III (SCIM).^9^,^12^,^23^,^24^ This revealed that higher UEMS and prehension were positively correlated with the percentage of time spent interacting (*Perc*), while higher SCIM and sensation scores were correlated with a greater number of interactions performed during the recordings (*Num*).^9^

These findings established the egocentric video-based technology as a valid tool for monitoring UE function at home. *Perc* and *Num* can be further exploited as outcome measures of hand function during daily life. In addition to the role of this technology as a tool for clinical research, it may have benefits in the context of outpatient rehabilitation, with the goal of remotely monitoring the progress of rehabilitation and fine-tuning the interventions according to the patient’s progress.^25^ To achieve this goal, the large amount of information produced by the monitoring system must be summarized and reported to clinicians in a usable way in order to promote the development of novel and optimized therapies for people with cSCI living in the community.

In this context, a plethora of mobile applications and web-based interfaces have been developed and released on the market or for research purposes, particularly for monitoring and delivering rehabilitative interventions.^26^-^28^ Several applications were developed for hand and UE rehabilitation, both for assessment and treatment purposes.^29^-^33^ However, most of these applications extracted and reported kinematic information of hand and finger movements (e.g., joint angles, range of motion, etc.) during specific tasks.^29^ To the best of our knowledge, none of the previous studies have developed an interface for reporting UE use captured during unconstrained activities recorded at home, which is essential for capturing the *performance* domain of hand function.

In the present study, we adopt user-centred design principles to develop a reporting interface and deliver simple egocentric video-based measures of hand function from the patients’ homes to clinicians. Our objectives were to determine the best way to report the information and to understand what pieces of information were more informative to different groups of healthcare professionals.

## Methods

This study was approved by the Research Ethics Boards at the University Health Network (UHN; Study #18-5232). We used a user-centred design approach to create a user-friendly clinical decision support (CDS) dashboard. This iterative design process started with focus groups to gather the perspectives of clinicians on what an optimal reporting format might look like. Our use of the term “reporting format” encompasses various elements, such as choice of metrics, visualization of metrics (e.g., tables, graphs), and choice of platform (e.g., print, web-based, mobile).

### Participants

Healthcare professionals (HCPs) were eligible if they had expertise in UE outpatient rehabilitation. A convenience sample of two male physical medicine and rehabilitation (PMR) physicians (i.e., physiatrists), three female occupational therapists (OT), and two female physiotherapists (PT) were recruited from the Toronto Rehabilitation Institute -UHN through word of mouth, team meetings, and clinical rounds. All participants were actively treating adults living with cSCI at UHN in a clinical program that specializes in cSCI care. All participants were assigned an alphanumeric code to anonymize transcript data and any excerpts in this article.

### User-Centred Design Process

In the initial phase of our user-centred design process, we randomly divided HCPs into two groups to have at least one representative of each profession in each group. Specifically, the first group was composed of 1 PMR, 1 PT, and 1 OT, whereas the second group was composed of 1 PMR, 1 PT, and 2 OTs. We conducted 45-minute focus group meetings with each group, facilitated by a semi-structured interview guide. We initially used focus groups to develop a broad understanding of the HCPs’ perceptions on the viability of egocentric cameras for monitoring hand use at home and to identify the kind of information we could extract from video footage to generate clinically useful summary reports. The interview guide for these sessions is provided in the **Appendix**. Insights from the focus groups were used to develop an initial low-fidelity prototype (i.e., a simple and rough representation of a design concept with limited functionality).

Following the focus groups, we transitioned to several rounds of one-on-one user interviews to capture a more in-depth and personalized understanding of clinician needs that may not have been fully voiced in a focus group setting. These discussions often revolved around explaining how we incorporated previous feedback, refining the metrics presented on the dashboard, and determining whether they were meaningful and reflected the useful parts of sample videos or how they could integrate this information into their existing workflow. The low-fidelity prototype was refined through these rounds of interviews, incorporating feedback with each round to ensure the evolving design was aligning with user needs and expectations. To ensure the accuracy of these changes during the iterative design process, users would confirm their feedback was correctly implemented during subsequent interviews, a strategy known as member checking. This process of refinement continued until the point of saturation where the information gathered from interviews no longer yielded new insights. The outcome of this iterative design process was a final, high-fidelity prototype (i.e., a more detailed prototype closely resembling the final product in both visuals and functionality).

All focus groups and interviews were recorded and transcribed verbatim. Interview data were analyzed using affinity diagramming.^34^ Transcripts of the interviews were read and reread for data immersion. Post-it notes were used to capture interview notes, facts, and observations. Notes were clustered according to similarity, with new clusters emerging from the data as needed. Each cluster formed a subtheme, and similar subthemes were grouped to identify overarching themes in the data. We created personas based on identified differences between HCPs and their needs or requirements.^35^ User stories were developed for each persona from the data and mapped to specific features to inform the development of the high-fidelity prototype.^36^

The iterative design process, along with the continual member checking, ensured the trustworthiness of our dashboard. The regular, documented checks allowed us to align the dashboard design with the needs of the users and offered us an ongoing assessment of its utility from the end-user’s perspective.

## Results

### Focus groups and interviews

A total of three iteration cycles were conducted. HCP feedback on the design of the CDS dashboard informed several changes to the low-fidelity prototypes over the course of the design process, such as the inclusion of graphs to display the temporal trends in measures of hand function and video snippets to observe the quality of hand use. We learned that HCP preferences varied; PTs and OTs favoured qualitative measures such as movement quality and patient video snippets, whereas PMRs preferred quantitative metrics to help motivate their patients to exercise. Four themes and 11 subthemes were identified through affinity diagramming (**Table 1**).

**Table 1.** Themes and subthemes identified through affinity diagramming.

**Theme**	**Description**	**Subthemes**
(1) Dashboard summaries	HCPs often have time constraints to review information in the dashboard. It would be beneficial to reduce the time burden of interpreting data by summarizing insights.	(1a) time constraints (1b) interpretation of data
(2) Qualitative measures of hand use	HCPs stressed the need to be able to watch videos of patient hand use to capture qualitative measures of hand function like postures or movement quality and the need for a patient diary to understand patient feelings and frustrations.	(2a) seeing patient videos (2b) measuring movement postures and quality (2c) inclusion of patient diaries
(3) Providing more context	HCPs emphasized the need for additional context to interpret the graphs. They noted that comparing plots to norms in the general population, normalizing the data by activity, and refining recording protocols could help in this regard.	(3a) comparing to population norms (3b) normalizing by activity or task (3c) patient biases
(4) Longitudinal goal / outcome tracking	HCPs identified several use cases for longitudinal tracking, including the ability to provide feedback outside the clinic, clinic outcomes tracking, and tailoring care for the patient’s home environment.	(4a) providing real-life feedback (4b) long-term outcome tracking (4c) adapting care to home environments

Overall, HCPs expressed a positive sentiment toward the CDS dashboard, recognizing its potential to monitor rehabilitation progress remotely, provide both quantitative and qualitative feedback on hand use in the patient’s own environment, fine-tune interventions based on home progress, and visually track progress over time to incentivize continued therapy at home. HCPs also expressed interest in developing a patient dashboard to allow patients to upload a diary entry or satisfaction scale with each video, track their own progress, and bolster motivation. They noted that this, along with options to filter the presented information by specific activities being performed, would supplement their understanding of patients’ progress and could help them identify factors contributing to changes in hand use. For example, if “there’s less interactions per hour, […] is there a new experience of pain? Did they overdo it the day before?” [OT1].

HCPs raised some concerns surrounding the implementation and use of the CDS dashboard. One participant noted:

It’s really hard to implement new technologies in clinical practice because many therapists might not be comfortable with it. […] maybe like a face-to-face training, so [the practice leads are] very familiar, and then they can train the other therapists. Because when it comes from the practice lead, I feel like more therapists are open to using it. [OT3]

They also indicated that adoption would be determined by “how easy it was [to use], because we have a very short amount of time with patients […] it would just have to be really quick” [PT1]. Therefore, some HCPs were in favour of having someone else interpret the results and display those summaries on the dashboard so they would not have to spend valuable appointment time going over the results and could instead focus on fine-tuning their interventions accordingly. As one participant explained, “An analogy would be if I send a patient for an MRI, […] I could look at the radiologist’s report” [PMR1].

### User stories and feature mapping

**Figure 1.**
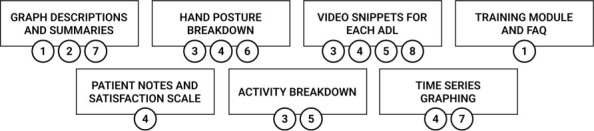
Mapping between user stories (IDs are found in Table 2) and dashboard interface features.

Based on our initial findings from affinity diagramming, our team employed user personas and user stories as tools to formalize user requirements for the high-fidelity prototype. The personas included for user story development were “therapist” and “physiatrist.” These personas were motivated by differences in clinician preferences that emerged during the interviews; PTs and OTs (therapist persona) favoured qualitative measures such as movement quality and patient video snippets, whereas PMRs (physiatrist persona) preferred quantitative measures to motivate patients. User stories were developed from the themes and subthemes identified in the affinity diagram and followed the format of “As a <persona>, I <want to>, <so that>.” (**Table 2**). User stories were then mapped to specific features discussed during interviews to inform the development of high-fidelity prototypes and deployment of dashboards in the future (**Figure 1**).

**Table 2.** User story development process

**(ID) User Story**	Theme	Supporting Quotes
(1) As a <therapist/ physiatrist>, I <want to be trained in how to use and interpret the data>, <so that I feel comfortable using it with patients>.	1b	*“It’s really hard to implement new technologies in clinical practice because many therapists might not be comfortable with it. […] maybe like a face-to-facetraining.”* [OT3]“ *The interpretation and understanding the implications of the data into their functions is something that is a disadvantage, that the clinicians should be aware of how to use this data.”* [PMR2]
(2) As a <therapist/ physiatrist>, I <want to see a summative evaluation of the data>, <so that I can grasp the information quickly>.	1a	*“I would want someone else to provide an interpretation. Like an analogy would be if I send a patient for an MRI, […] I could look at the radiologist’s report.”* [PMR1] *“Present it in a way that you can read it pretty quickly [..] if there’s some days, they’re not using their one hand, less or more, whatever the inconsistency is I’d like that to be highlighted.”* [OT3]
(3) As a <therapist>, I <want to see the quality of movement>, <so that I know my therapy is working since I can see how patients accomplished tasks>.	2a/b	*“I think that the quality of movement is important, especially as a PT, we are interested in the patient accomplishing the goal, but we also want to know how they accomplished it.”* [PT2] *“This would be helpful if I’m to know if my therapy is working […] because it’s the qualitative information that I need to see.”* [PT1]
(4) As a <therapist>, I <want to see external factors influencing hand use>, <so that I understand trends in the data>.	2c	*“I’d like to know why day eight, he’s used his left hand more. […] And then at the end of the month, he used his left hand equally with his right hand. What’s changed?”* [OT2] *“If their goal is ‘I want to be able to use my hands more for cooking.’ Do they spend more time cooking and are they satisfied with it? And then you can understand why they decrease or increase, if they’re meaningful, because in some people, an increase in time will be good and in other people, a decrease will be good, right?”* [OT1]
(5) As a <therapist/ physiatrist>, I <want information grouped by task or activity>, <so that the data has context and I can observe patient trends over time>.	3a/c	*“If you segmented it by activity, […] I want to see two examples of when this person was writing, and you watch two 20-second clips. That would help….”* [PMR1] *“This may just represent that they got better at turning on the camera when they knew they were about to use their hands.”* [PMR1] *“If there’s no context, it just doesn’t mean much, right? Because just in relation to one task, and then if that changes over time, […] even if you don’t have the quality piece, it still tells you something, right?”* [PT2]
(6) As a <therapist>, I <want to see norms for healthy populations>, <so that I can better optimize patient care towards those levels>.	3b	*“Norms where, you know, the normal population uses power sphere, for self-care 90% of time, but I see you only use it 5.2% of the time, but let’s try to improve that.”* [OT2] *“We don’t know compared to normative data. […] in a right-handed individual, what would this look like in the same one-hour period? […] Because no one’s doing the exact same activity.”* [PT1]
(7) As a <physiatrist>, I <want to see numerical trends>, <so that I can motivate patients and see the effect of treatment on our patient population long-term>.	4b	*“I think if we knew the patients had increasing use of their arms over time, they will be less reliant on caregivers and others. So I think that the overall use is important.”* [PMR1] *“And you have the ability to extract that into numbers and data, that will be more useful. […] Anything that is visual, works better with patients.”* [PMR2]
(8) As a <therapist>, I <want to see patient videos>, <so that I can provide real life feedback in their home environment or remotely administer and rate functional assessments>.	4a/c	*“It’s also giving us more ideas for what is difficult, what isn’t, what sometimes people can’t think about until they’re in their own environment.”* [PT1] *“We can do a feedback session with the patient and say, ‘See what you’re doing there? […] if you just do this with your thumb first or your wrist first….’ [I]t’s an opportunity for real life feedback.”* [OT1]

### High-fidelity prototype

**Figure 2.**
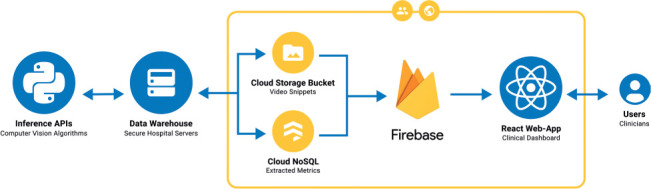
System architecture diagram for high-fidelity CDS dashboard.

The application was developed using React and JavaScript for the front end, Firebase for hosting and data storage, and Python for video processing (**Figure 2**). For long-term storage, all patient videos are saved in a data warehouse (i.e., secure servers at our research institution). Inference APIs process these videos to extract metrics to be reported to clinicians and stored in the data warehouse. Metrics and video snippets are uploaded in batches to the Cloud NoSQL database and Cloud Storage bucket, respectively. The clinical dashboard, developed using React and JavaScript, is hosted on Firebase and has access to the stored metrics and video snippets via the Firebase Database API.

**Figure 3.**
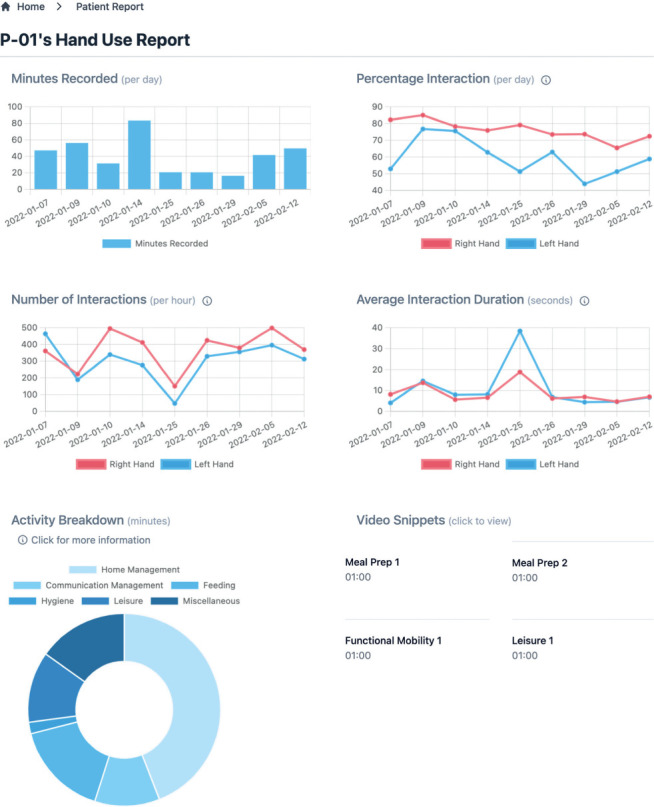
High-fidelity CDS dashboard prototype displaying mock data for illustration purposes. Features include time series graphing to monitor trends for metrics, descriptions for each metric, video snippets to qualitatively assess movement quality, and activity breakdown to provide hand-use context.

Our resulting high-fidelity prototype includes a secure login page, patient list, and corresponding dashboard for each patient. After logging in, the user is directed to the *Patient List* screen, where patients belonging to the authenticated user can be searched by name, clinic, or site of injury. Selecting a patient takes the user to the *Patient Dashboard*, where they can see the quantitative metrics extracted from patient videos, a breakdown of the activities performed in the videos, and video snippets representative of hand use for different recorded activities. Our high-fidelity prototype (**Figure 3**) does not yet include hand posture breakdowns, graph summaries, or a patient portal for video upload with diary entries and satisfaction scales. These features are subject to future development efforts.

## Discussion

This study describes the user-centred development of a dashboard to summarize and report information from wearable cameras worn by individuals with cSCI living in the community to clinicians. The study involved conducting focus groups and interviews with PMRs, PTs, and OTs to determine the best way to summarize and report information from the wearable cameras to ensure their usefulness in clinical practice. The design process involved three iteration cycles and incorporated clinician feedback to refine the interface.

HCPs expressed positive sentiments regarding the dashboard; they recognized its potential to monitor rehabilitation progress remotely, provide both quantitative and qualitative feedback on hand use in the patient’s own environment, and visually track progress over time. They also expressed interest in developing a patient dashboard to allow patients to upload a diary entry or satisfaction scale with each video and track their own progress, which could help identify factors contributing to changes in hand use. However, concerns were raised about the implementation of the dashboard and wearable cameras in clinical practice. Specific concerns, such as the interpretation of the dashboard, anxiety with using new technologies without the practice lead’s advocacy, and the time required for use of the dashboard, align with existing implementation science literature on factors influencing the successful translation of new tools in healthcare environments.^37^ Therefore, additional efforts should be made to better integrate the dashboard into the existing clinical workflow, provide onsite onboarding and ongoing technical support, and identify clinician champions to support implementation. This feedback reinforces the importance of clearly defining outcomes (e.g., feasibility and fidelity) and maintaining a broad view of the barriers and facilitators at multiple stages throughout the design and implementation of new healthcare tools.^38^,^39^

Our findings corroborate the importance of involving end-users in the design process and the potential benefits of web-based interfaces in remote monitoring of the rehabilitation progress of individuals with UE impairments. Previous work has shown that identifying the needs of end-users is crucial for adoption and for providing contextually relevant information and that web-based interfaces can provide richer insights into patient progress, promote home training, and enable patient-specific interventions to maximize motor recovery.^31^-^33^,^40^-^43^ However, the majority of previous work is focused on either assessment or training.^28^,^44^ To our knowledge, this is the first web-based interface that captures unconstrained hand use at home and reports measures of hand performance to clinicians. Therefore, successful implementation in clinical practice requires careful consideration of the needs and concerns of end-users and ongoing support to ensure clinician comfort during use.

Agile software development utilizes user stories to capture requirements.^43^ The user story template “as a <persona>, I <want to>, <so that>” enables stakeholders to discuss each requirement in detail and break them down into manageable pieces. This process ensures a shared understanding of end-user expectations, allowing software teams to build the right software.^45^ Our present work used this approach to identify clinician needs and requirements, which led to the identification of key elements of an interface that can better understand hand use at home after cSCI, such as descriptions and summaries to interpret graphs more efficiently, patient notes to understand patient satisfaction or struggles, activity breakdown to provide additional context, and time series graphing of metrics to track trends over time. Incorporating these elements can be generalizable to future development efforts aimed at capturing hand performance at home and serve as a starting point for any further refinement required by specific deployments or integration efforts in other rehabilitation settings.

Although this study provides valuable insights into the development of a reporting interface for egocentric measures of hand function in individuals with cSCI, there are several limitations to consider. One limitation is our lack of control for the influence of sex/gender on the participants’ feedback. Our focus was on collecting data based on the professional expertise of our participants, and the limited number of available staff in this single-site study precluded further stratification. Sex/gender of HCPs could potentially have influenced their experiences and expectations regarding the dashboard and should be considered in future studies. The small sample size of only seven clinicians from one hospital network may limit the generalizability of the findings to a larger population of clinicians and patients. However, regardless of sample size, clinical workflow analysis will likely be necessary to ensure minimal disruption to existing workflows and maximize adoption in future rehabilitation centers. Additionally, the study did not compare the developed reporting interface with other reporting formats. HCPs were presented with screenshots of the potential interface during interviews and focus groups, but they were only asked about different reporting formats without being presented with mock-ups of different formats, such as paper reports, tablets, mobile apps, or web apps. Presenting clinicians with mockups of different formats may have yielded a better understanding of the interface’s effectiveness. It is important to note that we designed this interface to report measures of hand function to HCPs in a theoretical environment. Reporting these insights to patients, families, or caregivers would require tailoring the dashboard for those specific user groups. Future work should pilot this interface to evaluate its usability, clinical utility, and effectiveness in real-world settings. Lastly, the outputs of this study could enable other applications of egocentric video at home, such as assessing falls risk during the transition between inpatient to outpatient care.

## Conclusion

The recovery of UE function in individuals with cSCI is a top priority, but several challenges impede optimal recovery, including premature discharge from inpatient care, poor monitoring of patient recovery in the community, and a lack of hand function outcome measures for assessing performance at home. To overcome these challenges, we utilized a user-centred design approach to create a reporting interface and deliver simple, egocentric video-based measures of hand function from patients’ homes to outpatient clinicians. Our results demonstrate that involving end-users in the design process and breaking down user requirements into user stories allowed us to identify the necessary interface elements for reporting hand performance metrics to clinicians. We also established the dashboard’s potential for remote monitoring of rehabilitation progress and motivating individuals with cSCI to continue their therapies at home. However, further inquiry is required to determine its usability, feasibility, and usefulness in real-world clinical practice.

## APPENDIX

### Focus Group Interview Guide

1. What are your thoughts about the clinical applications of using a wearable camera to observe (or to be observed) on how someone with an SCI functions in their home?
  - What types of benefits do you think it holds?
  - What types of challenges do you foresee with it?
  - How do you think it differs from traditional clinical assessments you have undergone (or have performed with clients)?
2. One of the things we are working on is to try to make the video data collection process as anonymous as possible so that people aren’t seeing everything that is going on. Here are some examples of the type of information that is obtained and “masked” from wearable camera videos of upper limb activities. When you see masked data output like this, what your initial impressions? [show video examples + output]
  - How would you rate your ability to understand this information in this sheet? What is clear to you? What isn’t clear and why?
  - What advantages do you over getting information this way versus being able to see the “raw video footage”? What concerns do you have with using raw footage versus this “masked” data’?
3. What types of data or information would be clinically meaningful to you?
  - What other types of formats would you like to see to help you better understand and use the information? [Probes here will include discussion of different platforms, metrics, and visualizations.]
  - What other types of other similar technology have you used? What did you like or dislike about those?
4. How would you incorporate it into your clinical practice?
  - When would you need to have access to this data in order for it to make a difference in the care that you deliver? How would you see it being integrated into your workflow?
  - What factors might influence your ability to use and receive this information (e.g., institutional issues – privacy, technological support, etc.)?
  - What factors might influence the ability to send this information (e.g., what about the person’s environment is needed to effectively set it up and send)?
5. Is there anything about wearable cameras that we didn’t discuss that you would like to share before we end?

### Follow-Up Interview Guide

1. Describe to participant how we have incorporated their previous feedback (e.g., show them examples of new system outputs or report formats, and point out how they have changed since the last iteration).
2. Explain to the participant that we will go over similar questions to the last meeting, but that we would like them to answer based on the newer version of the reports, which we have just shown them.
3. Use the following discussion questions (all or only some of these questions may be used, depending on which ones had outstanding issues in the last iteration):
  - Do you think that information obtained from wearable camera videos of upper limb activities would be useful to you? If so, how?
  - [Show example videos and system outputs] Do you think that these numbers are reflecting the most useful parts of the video? Do they make sense to you? What would you suggest instead?
  - Considering our earlier discussion about how you might use this information, how should the data be presented to you to make those applications possible? [Probes here will include discussion of different platforms, metrics, and visualizations.]
  - When would you need to have access to this data in order for it to make a difference in the care that you deliver? How would you see it being integrated into your workflow?
  - Are there factors that would prevent you from making use of this information?
